# Evaluating the Effect of Sumac Extract on Dentine Micro-Hardness during pH Cycling

**DOI:** 10.30476/dentjods.2022.92780.1677

**Published:** 2023-06-01

**Authors:** Mohammad Amin Akbari, Mohammad Bagher Rezvani, Mahshid Mohammadibasir, Mehrdad Karimi, Azadeh Balalai, Faeze Hamze, Seyed Abbas Hasheminejad

**Affiliations:** 1 Private Practice, Tehran, Iran; 2 Dept. of Operative, Shahed Dental School, Shahed University, Tehran, Iran; 3 Dept. of Traditional Medicine, School of Persian medicine, Tehran University of Medical Sciences, Tehran, Iran

**Keywords:** Sumac, Grape seed extract, Collagen, Dentine, Hardness

## Abstract

**Statement of the Problem:**

Although sumac extract (SE) is reported as a collagen cross linker, the available data regarding its effect on the dentine micro-hardness is quite sparse.

**Purpose:**

Therefore, the aim of this study includes evaluating the effect of different concentrations of SE on dentine micro-hardness comparing to grape seed extract (GSE).

**Materials and Method:**

In this experimental study, the GSE was purchased from available market and convert to 5% solution. Meanwhile the 5, 10, and 20% of SE solutions were prepared experimentally. The base line micro-hardness of 60 samples (30 premolars divided to buccal and lingual segments) was recorded triplicate for each specimen and they were randomly divided into 5 groups (four abovementioned experimental solutions and de-ionized water as negative control). For 35 consecutive days, each sample was twice pH cycled and treated by solutions. Ultimately, the final micro-hardness was recorded triplicate again for each sample and the numerical data was compared with each other using one-way ANOVA and Tukey HSD Post Hoc tests (α=0.05).

**Results:**

The meanSD values of micro-hardness for the groups was recorded as 54.45 13.4, 65.6518.5, 39.572.26, 41.131.66 and 43.794.96 at base line and 10.40.99, 11.85 0.75, 10.161.84, 8.481.16 and 6.311.01 at final stage for control, GSE 5%, SE 5%, SE 10% and SE 20% respectively. There was no significant difference among the micro-hardness of the groups before treatment (*p*= 0.369). However, after experimental treatment, there was significant difference between the groups (*p*= 0.024) while in pairwise comparison just two groups (GSE 5% and SE 20%) had significant difference with each other (*p*= 0.017).

**Conclusion:**

The efficacy of SE was reversely related to its concentration. Moreover, neither GSE nor SE had significant effect on dentine micro-hardness after 35 day pH cycling.

## Introduction

Bio-modification of dentine for improving dentine adhesion has been extensively surveyed and many research have been conducted to reach long survival for resin-infiltrated dentine over time [ 1 - 2 ].

Collagen cross linkers are now widely used in modern dentistry due to their beneficial effect on dentine-adhesive interface [ 3 - 4 ]. In fact, these agents could enhance the intra- and inter-molecular cross-links of collagen by promoting the hydrogen and/or covalent bonds [ 5 - 8 ]. These crossover bonds can prevent the long rod-like helical collagen from passing through each other under mechanical stress, which lead to more stiffness of dentine substrate [ 2 , 5 - 8 ]. Moreover, some of them would also inhibit the hazardous endogen enzymes such as matrix metalloproteinase (MMP) [ 9 - 10 ]. 

Several collagen cross linkers are introduced in literatures among which the synthetic gluteraldehyde is the most famous one [ 11 - 12 ]. Meanwhile, its application has been limited due to its cytotoxicity [ 1 ]. 

However, in recent years, incorporation of natural products as collagen cross linkers was widely investigated and their positive effect has been frequently reported [ 13 - 16 ]. Since natural extracts are often economic and safe, they are very favorable [ 17 - 18 ]. 

Among these natural gifts, the proanthocyanidin, which could be derived from different herbs, has been repeatedly documented as an effective natural collagen cross linker [ 19 - 21 ]. Grape seed extract (GSE) is one of the main sources enriched in proanthocyanidin. Therefore, the GSE has been vastly incorporated as dentine bio-modifiers for either collagen cross-linking or MMP inhibition [ 2 , 4 , 22 - 23 ]. 

Sumac is another helpful natural product. This plant grows mostly wild in the Mediterranean to the Middle East region [ 4 ] and it is considered as a quite strong antioxidant agent [ 24 ]. In addition, sumac has been introduced as a rich source of hydrolyzable tannins, a polyphenol compound that has the potency of cross linker agent [ 25 ].

The cross linkage of dentine collagen would enhance its mechanical properties [ 26 ], which could be manifested as increasing the micro-hardness. Actually, since the caries progress would lead to decreasing the micro hardness of dentine due to softening of collagen substrate, its enhancement could be beneficial [ 27 ]. Accordingly, some investigators reported that the GSE could increase the dentine micro hardness and their microscopic finding has confirmed their results [ 27 - 28 ].

Previous studies incorporated the micro-hardness test as an indicator for the efficacy of collagen cross-linkers in dentine [ 29 ]. However, the available data comparing the effect of sumac and grape seed on the micro-hardness of dentine is quite sparse. Therefore, the aim of this study includes evaluating the effect of different concentrations of sumac on dentine micro-hardness comparing to proanthocyanidin extracted from the grape seed. 

The null hypothesis stated that neither GSE nor sumac could increase the microharness of de-mineralized dentine samples and their effect would be similar to deionized water. 

## Materials and Method

### Grape Seed Extract (GSE)

An available GSE was purchased (Santa Cruz biotechnology, Dallas,TX, USA) and diluted to 5 wt.% solution by adding ethanol. Therefore the GSE 5% solutions was the only proanthocyanidin solution that was compared to different solutions of sumac.

### Sumac Extract (SE)

An amount of 500g of sumac was milled and sieved through 250m holes. The obtained powder was immersed in 2 lit of 80% ethanol and was shaken for 24h at room temperature. Thereafter, the whole solution was passed through #42 Whatman paper pouring into a petri-dish and stored at 45C in order to evaporate the extra ethanol. Finally, the remained material was considered as pure sumac extract (SE) that was mixed again with ethanol to produce three different solutions as 5, 10 and 20 wt.% SE.

### Artificial saliva

For each 500 ml of this artificial saliva, we mixed NaCl (21.9 g), CaCl_2_ (0.12 g), NaH_2_PO_4_ (0.13 g), and 5 ml of NaN_3_ (2%) with de-ionized water (Merck-KGaA, Darmstadt, Germany). The final pH of 6.5-7 was recorded for our artificial saliva.

### Demineralizing solution

For each 500 ml of this solution, we mixed NaCl (21.9 g), CaCl_2_ (0.12g), NaH_2_PO_4_ (0.13g), 5ml of NaN_3_ (2%), and 1.5cc of CH_3_COOH (Merck-KGaA., Darmstadt, Germany) with de-ionized water [ 30 - 32 ]. The final pH of 4-4.5 was recorded for our demineralizing solution. 

### Tooth preparation

In this study, we selected thirty intact human premolars, which were extracted for orthodontic reasons from patients with 18-25 years of age, all whom signed the informed consent. They were thoroughly examined and any cracked or decayed teeth were withdrawn. The samples were stored at 4°C in 0.9 %NaCl containing 0.02% NaN_3_ for prevention of any possible microbial contamination, and were used within six months after extraction.

Afterward, the teeth were sectioned mesio-distally by a low speed hand piece (Saeshin Precision Co., LTD. Daegu, Korea) that lead to duplication of samples to buccal or lingual segments (n=60). Each sample was mounted in epoxy resin while its un-ground surface was exposed. The buccal or lingual tooth surface were polished using silicon carbide papers (60, 240, 600 and 1000 grit respectively) by a polishing machine (Chennai Metco Pvt LTD., Chennai, India) and water spray. The polishing process was continued until the dentine was exposed (at least an oval 2×3 mm window was exposed in dentine). 

### Baseline micro-hardness

The baseline Vickers micro-hardness (Bareiss Prufgeratebau Inc.Oberdischingen, Germany) was recorded at least 100 µm distant from the DEJ using a diamond indenter by 1min/mm cross head speed and 490/03 mN load for 15 sec. 

This process was triplicate for each sample and the average was recorded as the baseline micro-hardness value of each specimen. 

### Experimental treatment

The 60 specimens were randomly divided into 5 groups including (1) GSE 5%, (2) SE 5%, (3) SE 10%, (4) SE 20%, and (5) Control: de-ionized water served as negative control [ 4 ].

**Figure 1:**
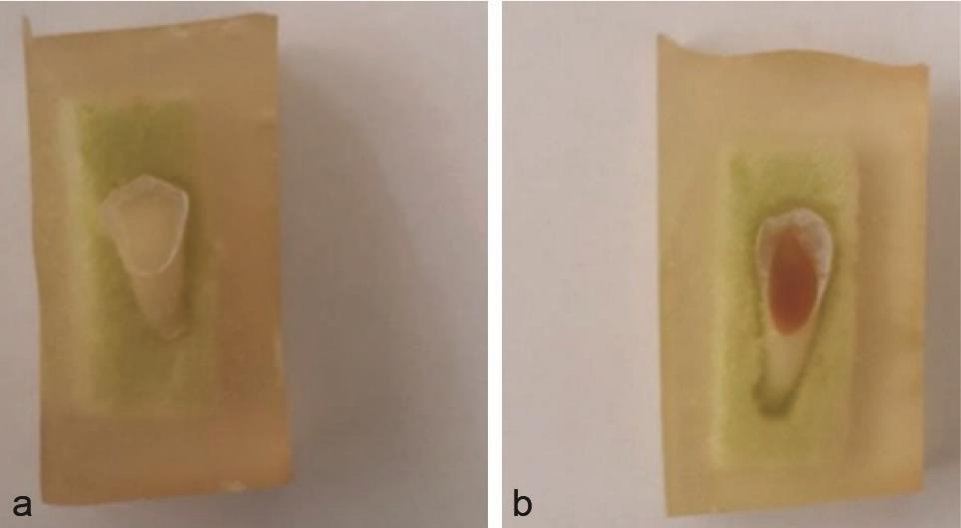
Mounting of the specimen and grounding the buccal or lingual surface to expose an at least 2×3 mm oval area in dentine: a: before treatment, b: after treatment

Each sample was immersed for one hour in demineralizing solution, 2 min washed by de-ionized water, 1 min covered by any of the mentioned treatment solutions, washed again by de-ionized water, and finally dipped in artificial saliva for 11 h. These steps were repeated in that order every 12 h (twice a day) for 35 consecutive days. An example of the samples before and after treatment is demonstrated in Figure 1.

### Final micro-hardness

The final micro-hardness of each sample was triplicate recorded just the same as the baseline process while the average of these three records were considered as the final micro-hardness value of each specimen.

### Statistical Analysis

The obtained numerical data were analyzed using one- way ANOVA, and Tukey HSD Post Hoc tests (α= 0.05). It should be emphasized that the normal distribution was confirmed by Kolmogorov-Smirnov test.

## Results

**Figure 2:**
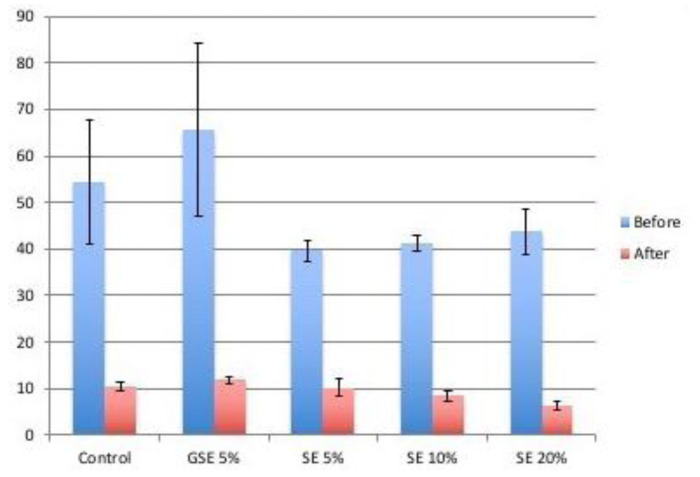
The micro-hardness mean ±S.D of different groups before and after treatment (SE: Sumac Extract, GSE: Grape Seed Extract)

The mean value of baseline and final micro-hardness± S.D of each group is demonstrated in Figure 2 and the descriptive statistics are presented in Table 1-2. One- way ANOVA analysis revealed no statistically significant difference between the baseline micro-hardness among the groups (*p*= 0.369). However, after treatment, the final micro-hardness of the experimental groups showed significant difference with each other (*p*= 0.024). As can be seen in Figure 2, the most final micro-hardness was recorded in GSE5% group and the least for SE20%. Furthermore, the Tukey HSD pairwise comparison showed that these two mentioned groups (GSE5% and SE20) had statistically significant difference with each other (0.017). In contrast, the other groups were not statistically distinguishable from each other. The pairwise comparison *P* values are represented in Table 3.

**Table 1:** Descriptive statistics of the groups, before treatment

	Mean	Std. Error	95% confidence interval for mean		Minimum	Maximum
			Lower Bound	Upper Bound		
Control	54.4528	13.40414	24.9505	83.9551	17.43	184.33
GSE* 5%	65.6528	18.50921	24.9143	106.3913	23.03	262.33
SE** 5%	39.5778	2.26495	34.5927	44.5629	28.97	51.27
SE 10%	41.1333	1.66560	37.4674	44.7993	33.60	48.67
SE 20%	43.7917	4.94284	32.9126	54.6708	19.13	80.50

* GSE: Grape seed extract

** SE: Sumac extract

**Table 2:** Descriptive statistics of the groups, after treatment

	Mean	Std. Error	95% confidence interval for mean		Minimum	Maximum
			Lower Bound	Upper Bound		
Control	10.4000	0.99654	8.2066	12.5934	2.17	14.77
GSE* 5%	118556	0.75515	10.1935	13.5176	7.30	16.43
SE** 5%	10.1611	1.84337	6.1039	14.2183	2.07	26.60
SE 10%	8.4806	1.16081	5.9256	11.0355	1.83	13.97
SE 20%	6.3167	1.01734	4.0775	8.5558	1.97	11.43

* GSE: Grape seed extract

** SE: Sumac extract

**Table 3:** The *P* value related to pairwise comparison of groups after treatment by experimental agents. SE: Sumac Extract, GSE: Grape Seed Extract

	Control	GSE* 5%	SE** 5%	SE 10%	SE 20%
Control		0.914	1.00	0.795	0.136
GSE 5%			0.859	0.295	0.017
SE 5%				0.863	0.180
SE 10%					0.715

* GSE: Grape seed extract

** SE: Sumac extract

## Discussion

Our results showed that GSE had better effect comparing to SE on micro-hardness of dentine exposed to demineralizing solution.

This finding is in accordance with Seseogullari-Dirihan *et al*. [ 4 ] who reported significant higher inhibition of total MMP activity for GSE comparing to SE. They also incorporated their experimental solutions for various time intervals and they argued that the best time for MMP inhibition was 1min [ 4 ], which is similar to our treatment time.

The MMP is existed in dentine substrate and would be activate during incorporation of acid etching that lead to failure of hybride layer in long term [ 33 ]. The effect of synthetic agents such as chlorhexidine for inhibiting these endogenous enzymes has been reported [ 33 ] that lead to enhancement of the bond durability [ 1 , 33 ]. However, some natural products such as GSE have been also proved to hinder the MMP that was quite beneficial for bond durability [ 34 ]. 

Similar to our results, regarding superior efficacy of GSE compared to SE, Seseogullari-Dirihan *et al*. [ 2 ] reported that the inhibitory effect of GSE against MMP was maintained even after 6-month incubation but the SE could not maintain its efficacy for that long period. 

The different concentrations of our solutions were in accordance to a previous study [ 4 ] while we examined three different concentrations for sumac. However, in both of the abovementioned studies [ 2 , 4 ], just one concentration of SE was examined. One of our interesting finding included that the beneficial efficacy of SE was reverse to its concentration. As it is demonstrated in Table 1, the GSE 5% group had no significant difference with neither of SE 5% nor SE 10% while it was significantly higher than SE 20%. The reverse effect of SE concentration on micro-hardness could be attributed to the pH of this solution, because the SE contains some acidic ingredients including malic, citric, gallic and ascorbic acids, that lead to low pH [ 35 ].

Nevertheless, an un-favorable result in our experiment included that there was no statistical significant difference between the negative control and neither of the treated groups. Although it could make doubt about the efficacy of both the GSE and SE, it is roughly in agreement with Epasinghe *et al*. [ 29 ]. Accordingly, they evaluated the micro-hardness of root surface and they found no statistical difference between the proanthocyanidin group and the negative control at 70-150 m depth [ 29 ]. On the other hand, it is in contrast with many other researches that investigated the inhibitory effect of proanthocyanidin and SE on MMP and argued the considerable beneficial effect of these two agents against MMP [ 2 , 4 ]. However, the study of Epasinghe *et al* [ 29 ] was more similar to ours because they also measured the micro-hardness.

In addition, we incorporate such longer pH cycling (35 days) comparing to other researchers (an average of 7-8 days) [ 36 - 37 ]. Therefore, this un-significant difference among control and treatment groups could be related to the long pH cycling time. For better decision, future studies with broader pH cycling time are strongly suggested to discuss about a threshold time interval for proanthocyanidin and SE efficacy on dentine. Since in theoretical aspects, both of these agents could bond to collagen, the optimal time threshold should be determined in future investigations.

Actually, about the detailed mechanism of action, both the GSE and the SE contains polyphenols [ 25 ]. Natural polyphenols could stabilize collagen polypeptides via the formation of multiple hydrogen bonds intra- or inter- long chains [ 25 ]. It has been documented that natural polyphenols are compounds of vegetable tannins and are present in fruits, nuts, vegetables, seeds, leaves, and flowers, such as grape seed, sumac berries, or curcumin [ 25 ]. Hence, either of these products could be considered in future studies. 

Although our investigation did not detect any significant beneficial effect for GSE and SE on the dentine micro-hardness, further studies are strongly suggested. Since one of the most important limitations of this study was lack of incorporating more concentration range of SE and GSE solutions, maybe expanding this concentration range may yield better results. Moreover, before clinical decision, definitely, the animal studies and clinical trials are required.

## Conclusion

Under the limitations of this study, the efficacy of SE was reversely related to its concentration. Moreover, neither GSE nor SE has shown significant effect on dentine micro-hardness after 35 day pH cycling.

## Conflict of Interest

The authors have no conflict of interests regarding this manuscript. 

## References

[1] Al‐Ammar A, Drummond JL, Bedran‐Russo AK ( 2009). The use of collagen cross‐linking agents to enhance dentin bond strength. J Biomed Mater Res Part B Appl Biomater.

[2] Seseogullari-Dirihan R, Mutluay MM, Pashley DH, Tezvergil-Mutluay A ( 2017). Is the inactivation of dentin proteases by crosslinkers reversible?. Dent Mater.

[3] Mazzoni A, Angeloni V, Apolonio FM, Scotti N, Tjäderhane L, Tezvergil-Mutluay A, et al ( 2013). Effect of carbodiimide (EDC) on the bond stability of etch-and-rinse adhesive systems. Dent Mater.

[4] Seseogullari-Dirihan R, Apollonio F, Mazzoni A, Tjaderhane L, Pashley D, Breschi L, et al ( 2016). Use of crosslinkers to inactivate dentin MMPs. Dent Mater.

[5] Bedran‐Russo AKB, Pereira PN, Duarte WR, Drummond JL, Yamauchi M ( 2007). Application of crosslinkers to dentin collagen enhances the ultimate tensile strength. J Biomed Mater Res Part B Appl Biomater.

[6] Tay FR, Pashley DH, Loushine RJ, Weller RN, Monticelli F, Osorio R ( 2006). Self-etching adhesives increase collagenolytic activity in radicular dentin. J Endod.

[7] Tezvergil-Mutluay A, Agee K, Uchiyama T, Imazato S, Mutluay M, Cadenaro M, et al ( 2011). The inhibitory effects of quaternary ammonium methacrylates on soluble and matrix-bound MMPs. J Dent Res.

[8] Tjäderhane L, Nascimento FD, Breschi L, Mazzoni A, Tersariol IL, Geraldeli S, et al ( 2013). Strategies to prevent hydrolytic degradation of the hybrid layer—a review. Dent Mater.

[9] Baena E, Cunha SR, Maravić T, Comba A, Paganelli F, Alessandri-Bonetti G, et al ( 2020). Effect of chitosan as a cross-linker on matrix metalloproteinase activity and bond stability with different adhesive systems. Mar Drugs.

[10] Balalaie A, Rezvani MB, Basir MM ( 2018). Dual function of proanthocyanidins as both MMP inhibitor and crosslinker in dentin biomodification: A literature review. Dent Mater J.

[11] Cai J, Palamara J, Burrow MF ( 2018). Effects of collagen crosslinkers on dentine: A literature review. Calcif Tissue Int.

[12] Lee J, Sabatini C ( 2017). Glutaraldehyde collagen cross‐linking stabilizes resin–dentin interfaces and reduces bond degradation. Eur J Oral Sci.

[13] Dávila-Sánchez A, Gutierrez MF, Bermudez JP, Méndez-Bauer L, Pulido C, Kiratzc F, et al ( 2021). Effects of Dentine Pretreatment Solutions Containing Flavonoids on the Resin Polymer-Dentine Interface Created Using a Modern Universal Adhesive. Polymers.

[14] De Carli G, Cecchin D, Ghinzelli KC, Souza MA, Vidal CdMP, Trevelin LT, et al ( 2018). Effect of natural collagen cross-linker concentration and application time on collagen biomodification and bond strengths of fiber posts to root dentin. Int J Adhes Adhes.

[15] Li FC, Kishen A ( 2018). Microtissue engineering root canal den-tine with crosslinked biopolymeric nanoparticles for mechanical stabilization. Int Endod J.

[16] Maravic T, Breschi L, Comba A, Cunha SR, Angeloni V, Nucci C, et al ( 2018). Experimental use of an acrolein-based primer as collagen cross-linker for dentine bonding. J Dent.

[17] Chen X, Daliri EBM, Kim N, Kim JR, Yoo D, Oh DH ( 2020). Microbial etiology and prevention of dental caries: exploiting natural products to inhibit cariogenic biofilms. Pathogens.

[18] Mulimani P ( 2017). Green dentistry: the art and science of sustainable practice. Br Dent J.

[19] Cai J, Burrow M, Manton D, Palamara J (2021). Using Proanthocyanidin as a Root Dentin Conditioner for GIC Restorations. J Dent Res.

[20] Hasanzadeh E, Mahmoodi N, Basiri A, Esmaeili Ranjbar F, Hassannejad Z, Ebrahimi-Barough S, et al ( 2020). Proanthocyanidin as a crosslinking agent for fibrin, collagen hydrogels and their composites with decellularized Wharton’s-jelly-extract for tissue engineering applications. J Bioact Compat Polym.

[21] Leme-Kraus A, Phansalkar R, Dos Reis M, Aydin B, Sousa A, Alania Y, et al ( 2020). Dimeric proanthocyanidins on the stability of dentin and adhesive biointerfaces. J Dent Res.

[22] Srivastava M, Yeluri R ( 2021). The effect of 10% alpha-tocopherol solution and 5% grape seed extract on the microhardness and shear bond strength to bleached dentin. Dent Res J.

[23] Wang Y, Liu Y, Liu H, Li S ( 2021). Dual-Functionality Evaluation of a Novel Collagen Crosslinking Resin. J Dent Res.

[24] Kosar M, Bozan B, Temelli F, Baser K ( 2007). Antioxidant activity and phenolic composition of sumac (Rhus coriaria L.) extracts. Food Chem.

[25] Seseogullari-Dirihan R, Mutluay M, Vallittu P, Pashley DH, Tezvergil-Mutluay A ( 2015). Effect of pretreatment with collagen crosslinkers on dentin protease activity. Dent Mater.

[26] Bedran‐Russo AKB, Pashley DH, Agee K, Drummond JL, Miescke KJ ( 2008). Changes in stiffness of demineralized dentin following application of collagen crosslinkers. J Biomed Mater Res Part B, Appl Biomater.

[27] Hussein F, Hashem SN, Elsayed SR ( 2021). The synergetic effect of silver diamine fluoride with potassium iodide and grape seed extract on dentin remineralization. Al-Azhar Dent J Girls.

[28] Yassen AA, Safy RK ( 2018). Grape seed extract and dentin remineralization. Egypt Dent J.

[29] Epasinghe DJ, Yiu CKY, Burrow MF, Hiraishi N, Tay FR ( 2013). The inhibitory effect of proanthocyanidin on soluble and collagen-bound proteases. J Dent.

[30] Ozgul BM, Orhan K, Oz FT ( 2015). Micro-computed tomographic analysis of progression of artificial enamel lesions in primary and permanent teeth after resin infiltration. J Oral Sci.

[31] Ten Cate J, Timmer K, Shariati M, Featherstone J ( 1988). Effect of timing of fluoride treatment on enamel de-and remineralization in vitro: a pH-cycling study. Caries Res.

[32] White D, Featherstone J ( 1987). A longitudinal microhardness analysis of fluoride dentifrice effects on lesion progression in vitro. Caries Res.

[33] Saffarpour A, Valizadeh S, Amini A, Kharazifard MJ, Rohaninasab M ( 2020). Effect of matrix metalloproteinase inhibitors on microtensile bond strength of dental composite restorations to dentin in use of an etch‐and‐rinse adhesive system. Clin Exp Dent Res.

[34] Sanon K, Sanchavanakit N, Srisawasdi S ( 2019). Grape seed extract reduces active gelatinases using an etch-and-rinse mode universal adhesive. J Adhes Dent.

[35] Fereidoonfar H, Salehi-Arjmand H, Khadivi A, Akramian M, Safdari L ( 2019). Chemical variation and antioxidant capacity of sumac (Rhus coriaria L.). Ind Crops Prod.

[36] Epasinghe D, Yiu C, Burrow M ( 2015). Synergistic effect of proanthocyanidin and CPP‐ACFP on remineralization of artificial root caries. Aust Dent J.

[37] Mai S, Kim YK, Kim J, Yiu CK, Ling J, Pashley DH, et al ( 2010). In vitro remineralization of severely compromised bonded dentin. J Dent Res.

